# The sacral autonomic outflow is parasympathetic: Langley got it right

**DOI:** 10.1007/s10286-018-0510-6

**Published:** 2018-02-16

**Authors:** John P. Horn

**Affiliations:** Department of Neurobiology and Center for Neuroscience, University of Pittsburgh School of Medicine, Pittsburgh, PA 15238 USA

**Keywords:** Taxonomy, Autonomic nervous system, Sympathetic, Sacral, Parasympathetic

## Abstract

A recent developmental study of gene expression by Espinosa-Medina, Brunet and colleagues sparked controversy by asserting a revised nomenclature for divisions of the autonomic motor system. Should we re-classify the sacral autonomic outflow as sympathetic, as now suggested, or does it rightly belong to the parasympathetic system, as defined by Langley nearly 100 years ago? Arguments for rejecting Espinosa-Medina, Brunet et al.’s scheme subsequently appeared in e-letters and brief reviews. A more recent commentary in this journal by Brunet and colleagues responded to these criticisms by labeling Langley’s scheme as a historical myth perpetuated by ignorance. In reaction to this heated exchange, I now examine both sides to the controversy, together with purported errors by the pioneers in the field. I then explain, once more, why the sacral outflow should remain known as parasympathetic, and outline suggestions for future experimentation to advance the understanding of cellular identity in the autonomic motor system.

## Introduction

The principles drawn from autonomic neuroscience are essential for understanding human physiology and the pathology of disease [20]. Due to the fundamental importance of autonomic physiology and pharmacology, the proposed renaming of the sacral autonomic outflow as sympathetic [10] would, if correct, force significant change in the concepts that drive biomedical research and clinical practice. For this reason alone, the new scheme requires serious consideration. In addition, one cannot ignore that the disruptive new idea came from the highly respected laboratory of Professor John-Francois Brunet in Paris and that it appeared in a prestigious, highly cited journal. The Brunet group’s radical conjecture triggered a wave of negative commentaries that enumerated strong factual arguments for rejecting change by maintaining the current definition for the sacral parasympathetic motor system [19, 24–27, 33]. A subsequent commentary in this journal [9] dug further into the history of the field in order to support the claim of scientific myth making. In my view, the dispute does not arise from conflicting experimental observations or deception, but instead from different interpretations of the evidence and from different readings of the field’s history.

## Core arguments for the Brunet conjecture

The key arguments that support the Brunet conjecture rest on the differential expression of transcription factors in mice, primarily on embryonic day 13.5 [10]. By comparing presumptive preganglionic neurons in the dorsal motor nucleus of the vagus (nX) with presumptive preganglionic neurons in the thoracic and sacral spinal cord, they detected *Phox2a*, *Tbx20, Tbx2 and Tbx3* in nX, but not in the thoracic or sacral cord. Conversely, *Foxp1* was detected in the thoracic and sacral cord, but not in nX. Based on the co-segregation of thoracic and sacral traits from cranial traits, it was concluded that thoracic and sacral pools of preganglionic neurons share a common sympathetic identity. Alternatively, another interpretation is possible. Thoracic and sacral preganglionic neurons may simply share a common spinal identity [33]. The same approach was also applied to assess the phenotypic identity of ganglionic neurons using transcription factors [10]. *Hmx2 and Hmx3* were detected in several cranial parasympathetic ganglia, but not in lumbar paravertebral sympathetic ganglia or in the pelvic ganglion. Conversely, the sympathetic ganglia and pelvic ganglion selectively express Islet1, Gata3 and Hand1. In addition, genetic deletion of *Olig2,* which disrupts formation of cranial parasympathetic ganglia [7, 8], failed to alter the size of the pelvic ganglion or the formation of sympathetic ganglia. Although one can interpret these observations in support of the Brunet conjecture [10], it is also possible that they simply reflect the segmental origin of different ganglia, rather than phenotypic neuronal identity as sympathetic or parasympathetic.

## Mythology versus insight

The Brunet group’s recent commentary [9] re-asserts that neurons in the sacral autonomic outflow should be defined by their “genetic make-up and dependencies” rather than by widely used classical criteria [20]. Pointing to the early history of the field, the commentary argues that the original designation of the sacral autonomic outflow occurred “in a remarkably cursory fashion, with a brief justification in 1899”. It goes on to point out errors in various anatomical schematics of the autonomic system, created between 1920 and 1949, as evidence for a mythology based on ignorance. The essay portrays the early literature as dogmatic and as motivated by a need to substantiate the dogma. No one would dispute that autonomic anatomy is complex, especially in the pelvic region, and that many studies overlook sexual dimorphism and that older literature and textbooks contain errors and oversimplifications. For a comprehensive contemporary overview of bladder control, see reviews by de Groat et al. [4, 5]. However, questioning the motives of scientists in a much earlier era seems not only dubious, but unlikely to shed light on today’s challenges. Moreover, it seems inappropriate to criticize the experimental approaches used before the advent of electrical recordings from nerves—this only became possible in the wake of World War I when vacuum tube technology led to the invention of electrical amplifiers and oscilloscopes [18]. Given the tools of the day—simple nerve stimulation and rudimentary pharmacology, coupled to simple observations of smooth muscle contractions, blood flow and glandular secretions—the accomplishments of Walter Gaskell and then John Langley are all the more remarkable. Did their data fully justify all their conclusions? No and certainly not by today’s standards. Instead of labeling this as myth building, it is perhaps more useful to think of it as deep insight informed by 50 years of careful, systematic experimental observation. Looking back to *Fin de siècle* neuroscience, Langley’s insight appears more akin to the imaginative ideas developed by Santiago Ramon y Cajal during his ground-breaking explorations of neuroanatomy. We can only hope that in 100 years, future neuroscientists will find something of enduring value in our early 21st century efforts, crude as they may be! Despite these cautions, I agree with the Brunet group that one should acknowledge the history of ideas as a prelude to incorporating genetic mechanisms into autonomic neuroscience.

## Origins of modern nomenclature for the peripheral autonomic system

To recount the history of autonomic neuroscience, one must acknowledge John Newport Langley (1852–1925) [6, 16]. Building on the work of Walter Gaskell and others, Langley introduced the concept of an autonomic system and the logic for dividing it into three divisions—sympathetic, parasympathetic and enteric. Apart from a brief visit to Heidelberg while a student, Langley spent his entire academic career in the physiological laboratories at the University of Cambridge, where he studied peripheral autonomic pathways and their effects upon target organs in amphibians, birds and mammals. In addition to exerting influence through his research, Langley edited and owned the Journal of Physiology from 1894 until his death. At the end of his career, Langley published an important monograph that sums up his life’s work and speculates about the importance of phylogeny and ontogeny for understanding how the nervous system is organized and functions [31]. In agreement with the Brunet group [9], I reject Langley’s archaic speculations on evolution and development. Instead, one should focus on the words and language in Langley’s monograph concerning functional divisions of the autonomic system, most of which remains remarkably clear nearly 100 years after publication.

Langley opens by recognizing Jacques-Benigne Winslow [42], an influential Professor of Anatomy and Surgery at the University of Paris, whose textbook of human anatomy describes the vagus and splanchnic nerves as sympathetic. This usage signified the bringing of internal organs into harmony and extended to all autonomic nerves, a notion that Langley rejects.

“Sympathetic nerves have no special relation to sympathies.” page 7 [31]

Langley introduced the concept of an “autonomic” nervous system in order to distinguish nerves that control smooth muscles and glands from the somatic motor nerves that control striated muscles. In choosing the term autonomic, Langley also sought to find a better word than “vegetative” and “involuntary”. He argued that vegetative implied a false relationship with plants and that involuntary was inadequate because people can initiate certain autonomic actions as a matter of will (e.g. changes in heart rate, tear production through crying). Despite this rationale, use of the term vegetative persists in some non-English speaking countries [23].

The decision to move beyond Winslow’s earlier usage by dividing the autonomic system into three divisions was essential in order to capture its organization and function.

“…the chief objection to calling the whole autonomic system sympathetic is that it confuses instead of simplifying nomenclature:” page 7 [31].

Ironically, the same logic applies today to Brunet’s conjecture. Reverting to a common name for the thoraco-lumbar and sacral autonomic outflows does not simplify discussion or understanding of the autonomic motor system because these elements display distinct features [25, 33].

## Gaps in the central outflow

Langley defined the sympathetic, parasympathetic and enteric systems using several criteria [31], beginning with their different central outflows. He noted that the enteric system operates exclusively within the gastro-intestinal tract and is relatively independent from central control. Today, all agree that the enteric system contains sensory neurons and interneurons in addition to motor neurons and that enteric circuits undergo inhibitory modulation by sympathetic motor pathways through prevertebral ganglia and splanchnic nerves and excitatory modulation by parasympathetic motor pathways through the vagus and sacral outflow [14, 23].

Today’s controversy, like most of Langley’s monograph [31] focuses on the distinctions between the sympathetic and parasympathetic divisions. The monograph notes that gaps exist in the central outflow of autonomic nerves at the levels of the limb enlargements. Between the limb enlargements, he calls the system sympathetic. Rostral and caudal components become parasympathetic because they are located beside the sympathetic region. Although all agree that these gaps exist [9, 10], Brunet’s group speculates that they are an unimportant consequence of limb motorneuron development that exhausts the local segmental pools of motor progenitor cells. I agree it would be interesting to investigate this conjecture and related hypotheses concerning the mechanistic origin of segmental gaps in the central autonomic outflow.

## Differences in targets and territories

The sympathetic and parasympathetic outflows differ in terms of their targets, the pathways of their peripheral nerves and their functional attributes. One can trace these concepts to Langley, together with the idea of functional opposition—they all remain deeply embedded as core principles of autonomic neuroscience.

“The facts that the sympathetic innervated the whole body, whilst the cranial and sacral outflows innervated parts only, and that the sympathetic had, in general, opposite functional effects from those of the other autonomic nerves, indicated that the sympathetic was distinct from the rest.” page 8 [31].

The following examples support Langley’s view, but are not explained by the Brunet conjecture.

- In general, the sympathetic system, but not the parasympathetic system (cranial and sacral), innervates the skin. An exception to this pattern has been reported in the lower lip of the cat [21, 22].
- Thermoregulation is exclusively sympathetic and arises primarily through thoraco-lumbar control of the cutaneous circulation, piloerection, sweat glands and brown fat.
- Blood pressure control operates primarily through sympathetic regulation of the cardiovascular system and kidneys.
- Vagal parasympathetic inhibition of the heart opposes sympathetic excitation.
- Sacral parasympathetic activation promotes micturition, while sympathetic activation promotes urine retention [4, 5].
- Sympathetic neurons in paravertebral chain ganglia are selectively innervated by thoraco-lumbar sympathetic preganglionic neurons, and not by sacral or suprasegmental parasympathetic preganglionic neurons. The axons of sacral preganglionic neurons never enter the paravertebral sympathetic chain.
- Sympathetic ganglia (paravertebral and prevertebral) are intimately associated with large arteries, while parasympathetic ganglia are often embedded within target organs (e.g., salivary glands, bladder). Brunet’s group takes issue with this interpretation [9], noting that the pelvic ganglion may not always be diffusely organized and that it often contains an apparent sympathetic component. They have a point, but are only partially correct. The anatomy of pelvic autonomic ganglia is complex and variable in different animals. For example, the pelvic ganglion in mice and rats tends to be discrete, but is broken into many mini-ganglia in the human [29].

## Neurotransmitter phenotype distinguishes between sympathetic and parasympathetic neurons

The recent papers from Brunet’s group [9, 10] correctly argue that the pharmacology and transmitter status of autonomic neurons is more intricate than often portrayed in textbooks. They point to cholinergic sympathetic neurons as evidence that transmitter status cannot serve as a criterion to define sympathetic and parasympathetic neurons. Langley was aware of this anomaly, but not its full explanation.

“The only structures markedly influenced by sympathetic stimulation which are not influenced by adrenaline after nerve section are the sweat glands of the cat and some other mammals.” page 29 [31]

By the 1930s it became clear that cholinergic sympathetic mediation of sweating was an exception to the rule that postganglionic sympathetic neurons use norepinephrine as their transmitter [3, 36]. The first step in understanding the developmental origin of cholinergic sympathetic neurons came through the discovery that environmental factors could switch the functional neurotransmitter status of rat sympathetic neurons in primary cell culture from noradrenergic to cholinergic [15, 34, 35]. Subsequent studies demonstrated that cholinergic sympathetic neurons innervate the periosteum as well as sweat glands [1]. Careful analysis showed that these neurons undergo a process of transdifferentiation in which they initially express a functional noradrenergic phenotype and then, under the influence of factors released by the sweat glands and periosteum, undergo a transition to a functional cholinergic phenotype [12]. This switch in transmitter status depends on signaling through the gp130 cytokine receptor [38]. It is important to note that this differs from cranial parasympathetic neurons, which sometimes express tyrosine hydroxylase, but do not synthesize detectable levels of norepinephrine [30, 32]. Tyrosine hydroxylase has also been detected in 5% of parasympathetic paracervical pelvic ganglion neurons, but the functional transmitter status of these cells remains unknown [28]. Together, these observations suggest that the transdifferentiation of cholinergic sympathetic neurons differs from the genesis of cholinergic parasympathetic neurons.

## Moving forward

The work from Professor Brunet’s laboratory serves an important purpose by illustrating the power of developmental molecular genetics to illuminate important features of the autonomic motor system. It should motivate us to re-examine long-held beliefs. Although the interesting results from their experiments do not justify a reclassification of the sacral parasympathetic as sympathetic, they point to a path forward. For the time being, I conclude that Langley got it right concerning the three divisions of the autonomic motor system. Moving forward, transcriptomic methods now make it possible to identify patterns of gene expression that characterize distinctions between populations of adult autonomic neurons [13]. This approach should strengthen future efforts to understand in molecular genetic terms the functional organization of the autonomic outflow and its developmental origins. Another important issue regards the development of functional subclasses of autonomic neurons that innervate blood vessels, different types of glands, brown fat and other targets. Although such phenotypic specializations begin to appear during ganglionic development [17, 39–41], the underlying mechanisms remain poorly understood [2, 37]. Coming to a deeper understanding of autonomic behavior will require solving the problem of neuronal identity in terms of multiple criteria based on molecular genetics, developmental origins, functional circuitry and neuronal activity [11]. Bringing together all these facets of autonomic neuroscience will provide the data required to test classical concepts and then build upon them. Basic and clinical autonomic neuroscientists should embrace such interactions and they should remain open to the possibility of change. If Langley were alive today, he might agree.
